# Effects of Own and Partner Pain on Spousal Caregivers’ Marital Quality

**DOI:** 10.1093/geroni/igab046.1151

**Published:** 2021-12-17

**Authors:** Suyoung Nah, Lynn Martire

**Affiliations:** The Pennsylvania State University, University Park, Pennsylvania, United States

## Abstract

Little is known about whether care recipients’ and their spousal caregivers’ own pain influence the marital quality perceived by caregivers. Considering that experiencing and witnessing pain may be related to marital distress, we hypothesized that care recipient and caregiver pain would be associated with caregivers’ greater increases in marital conflict over time. We focused on 264 spousal caregivers of older adults with chronic illnesses or disability from the 2015 and 2017 National Study of Caregiving. Sixty-nine percent of care recipients and 54% of caregivers in this study were bothered by pain at baseline. Findings revealed that caregiver (b = 0.25, p = .02) and care recipient pain (b = 0.34, p < .01) at baseline were both associated with caregivers’ higher marital conflict at follow-up. These findings suggest the importance of accounting for not only care recipients’ pain but also spousal caregivers’ own pain when examining caregivers’ marital quality.

